# LncRNA SLCO4A1-AS1 predicts poor prognosis and promotes proliferation and metastasis via the EGFR/MAPK pathway in colorectal cancer

**DOI:** 10.7150/ijbs.38041

**Published:** 2019-11-08

**Authors:** Rui Tang, Junhong Chen, Mengtian Tang, Zhiqiang Liao, Lianqing Zhou, Jiarui Jiang, Yingbin Hu, QianJin Liao, Wei Xiong, Yanyan Tang, Shaolin Nie

**Affiliations:** 1 Hunan Cancer Hospital and The Affiliated Cancer Hospital of Xiangya School of Medicine, Central South University, Changsha, China.; 2 The University of South China, Hengyang, China; 3 Department of Colorectal Surgery, Hunan Cancer Hospital and the Affiliated Cancer Hospital of Xiangya School of Medicine, Central South University, Changsha, China; 4 Central Laboratory, The Affiliated Cancer Hospital of Xiangya Medical School, Central South University, Changsha, China; 5 The Key Laboratory of Carcinogenesis and Cancer Invasion of the Chinese Ministry of Education, Cancer Research Institute, Central South University, Changsha, Hunan, China

**Keywords:** long non-coding RNA (lncRNA), SLCO4A1-AS1, poor prognosis, EGFR/MAPK, colorectal cancer (CRC)

## Abstract

It is universally acknowledged that long non-coding RNAs (lncRNAs) involved in tumorigenesis in human cancers. However, the function and mechanism of many lncRNAs in colorectal cancer (CRC) remain unclear. By analyzing the two sets of CRC-related gene microarrays data, downloaded from the Gene Expression Omnibus (GEO) database and the lncRNA expression in a set of RNA sequencing data, we found that lncRNA SLCO4A1-AS1 was significantly upregulated in CRC tissues. We then collected CRC tissue samples and verified that SLCO4A1-AS1 is highly expressed in CRC tissues. Furthermore, SLCO4A1-AS1 was also upregulated in the CRC cell line. *In situ* hybridization results showed that high expression of SLCO4A1-AS1 was associated with poor prognosis in patients with CRC. Next, we found that SLCO4A1-AS1 promoted CRC cell proliferation, migration, and invasion. Results of western blotting assays show that its mechanism may relate to the epidermal growth factor receptor (EGFR)/mitogen-activated protein kinase (MAPK) pathway. Therefore, SLCO4A1-AS1 may be a potential biomarker for CRC prognosis and a new target for colorectal cancer therapy.

## Introduction

Colorectal cancer (CRC) is the third most common cancer in the world^1^,^2^, also is the second leading cause of cancer-related death^3^. In 2018, more than 860,000 patients died of colorectal cancer all over the world, and the mortality rate is 8% to 9%^4^. Recently, with the changes in the Western diet, the incidence of colorectal cancer is increasing. Surgery is the most effective treatment for colorectal cancer^5^,^6^. Over 90% of CRC patient can be cured by surgery if diagnosed at an early stage^7^. With the increasing development of multidisciplinary, radiotherapy and chemotherapy, the survival and quality of life of patients with colorectal cancer have been improved. Unfortunately, patients with CRC are often diagnosed as advanced, with a poor prognosis and a lower 5-year survival rate^8^. In recent years, it has been found that long non-coding RNAs can be used as a new target for cancer diagnosis and treatment, and has important research value^9^-^13^.

Long non-coding RNAs (lncRNAs) is a kind of non-coding RNAs with more than 200 nucleotides in length^14^,^15^. Recently a variety of evidence has shown, lncRNAs play a crucial role in promoting tumors or suppressing tumors during tumorigenesis and development^16^-^19^. Meantime, they involved in the regulation of apoptosis, cellular differentiation, tumorigenesis and metastasis^20^. In addition, they also influence the growth of tumor cells through epigenetic regulation, regulate the expression of genes at the transcriptional and post-transcriptional levels^21^-^23^ or tightly associated with chromatin remodeling^24^, and thus play an essential role in many physiological processes (such as cancer), affecting the occurrence of tumors, development, invasion, and metastasis and relationship with prognosis^25^-^28^. What's more, recent studies have shown that tumorigenesis is associated with dysregulation of lncRNAs, and many studies have confirmed that lncRNAs play an important role in the pathogenesis of colorectal cancer^29^-^33^. In addition, lncRNAs expression can provide clinical information about tumor outcomes and can be used as a diagnostic or prognostic marker^34^-^39^. However, the clinical significance and biological mechanisms of lncRNAs in the progression of CRC remain largely unknown.

In this study, we downloaded two sets of CRC-related gene microarrays GSE32323 and GES39582 through the Gene Expression Omnibus (GEO) database and constructed a differential expression profile of lncRNA, and the RNA sequencing data set GSE104836 was downloaded to further compare with the gene microarrays data to analyze the differential expression of lncRNA in CRC. We screened lncRNA SLCO4A1-AS1 with up-regulated expression in two sets of gene microarrays and RNA sequencing data. Then we detected differential expression in fresh CRC tissues and several CRC cell lines by reverse transcription PCR (RT-PCR). Furthermore, *in situ* hybridization was used to detect the expression of SLCO4A1-AS1 in paraffin-embedded tissues. Next, we investigated the biological behavior of SLCO4A1-AS1 in CRC by inhibiting the expression of SLCO4A1-AS1 in CRC. Finally, Western Blotting proved that SLCO4A1-AS1 as an oncogene promotes the development of CRC and was related to the EGFR/MAPK pathway.

Overall, we have initially proved that lncRNA SLCO4A1-AS1 acts as an oncogene and promotes the development of colorectal cancer through the EGFR/MAPK pathway. Based on this study, SLCO4A1-AS1 may become a novel biomarker or therapeutic target for colorectal cancer in the future.

## Materials and methods

### Datasets analysis

Two sets of CRC-related gene microarrays GSE32323, GES39582 and RNA sequencing data set GSE104836 were obtained from the GEO database. Among them, GSE32323 contains 17 tumor tissues and 17 normal tissues, and GES39582 contains 433 tumor tissues and 19 normal tissues. We filtered the background noise from the gene expression profile and then analyzed the data using Significant Analysis of Microarray (SAM) software. The cut-off fold change value for differentially expressed lncRNA was set at ≥ 1.5-fold change and false discovery ratio (FDR) was < 0.05. The significant lncRNAs in GSE32323, GSE39582 were demonstrated by heat maps generated using Genesis software. GSE104836 contains 10 tumor tissues and 10 normal tissues. We use MORPHEUS, a kind of versatile matrix visualization and analysis software to view GSE104836 dataset as a heat map. https://software.broadinstitute.org/morpheus/.

### Clinical samples

Two sets of clinical tissue samples were collected in this study. The tissue samples used in the RT-PCR experiment were from the Affiliated Cancer Hospital of Central South University from 2017 to 2018, including 45 CRC tissues and 45 adjacent normal tissues, all from surgically removed specimens. The tissue samples are used in the *in situ* hybridization experiment were paraffin-embedded CRC tissue samples from 165 patients who underwent surgery from January 2009 to September 2012 in the Affiliated Cancer Hospital of Central South University. The clinicopathological data are shown in Supplementary Table 1. The study was approved by the Ethics Committee of the Affiliated Cancer Hospital of Central South University and each patient signed a written informed consent form.

### RNA isolation and qRT-PCR

Total RNAs were extracted using TRIzol reagent (Invitrogen, USA). One µg of total RNA from the samples was reverse transcribed using a Reverse Transcription Kit (BioRad, Hercules, CA, USA). RT-PCR was performed using SYBR Green (BioRad) in the LightCycler 480 RT-PCR Detection System (Roche). Primers were synthesized by Sangon Biotech Company (Shanghai, China): SLCO4A1-AS1 forward 5'-CACTTTCCAGCCTCTCACCA-3', and reverse 5'-GGCCACCTCCTCAAACAAGA-3'; β-actin forward 5'-TCACCAACTGGGACGACATG-3', and reverse 5'-GTCACCGGAGTCCATCACGAT-3'. SLCO 4A1-AS1 expression was normalized to the respective β-actin expression level. Relative expression was calculated using the equation: ΔCt = Ct (target gene) - Ct (β-actin), fold expression = 2^-(ΔCt(tumor) -^ ΔCt(normal)) by Cq value.

### *In situ* hybridization and scoring evaluation

*In situ* hybridization was performed to detect SLCO4A1-AS1 expression in tissue specimens using a nucleotide probe. The SLCO4A1-AS1 probe was designed and synthesized by Sangon Biotech Company (Shanghai, China): 5'-GGUCCUCUGCUUUUAUGUCAGUUCUCAGAAACAGAGUCUUCAAG-3', 5' labeled with GIG-dUTP tag.

The experiment was conducted according to the manufacturer protocol using the sensitive enhanced *in situ* hybridization kit of BOSTER Company (Wuhan, China). A semi-quantitative scoring standard of ISH was used in which the staining intensity and the number of positive regions were recorded^40^. The scoring was graded as 0 (negative), 1 (< 10% positive), 2 (10% - 50% positive), or 3 (> 50% positive) in accordance with the staining proportion and intensity. The final scores were regarded as low expression (0-1) and high expression (2-3). All sections were independently scored by two pathologists who were blinded to the clinicalpathological features and the clinical data.

### Cell line and gene silencing

The CRC cell lines HT29 and SW480 were maintained in an atmosphere of 5% CO_2_ at 37 °C and cultured in RPMI 1640 medium (FBS, BI) supplemented with 1% antibiotics (100U/ml penicillin and 100 µg/ml streptomycin sulfates) and 12% fetal bovine serum (FBS, ZETA). While the normal colorectal cell line NCM460 and CRC cell lines HCT116 were maintained in an atmosphere of 5% CO_2_ at 37°C and cultured in RPMI DMEM medium (FBS, BI) supplemented with 1% antibiotics (100U/ml penicillin and 100 µg/ml streptomycin sulfates) and 12% fetal bovine serum (FBS, ZETA).

For gene knockdown, cells were seeded in six-well plates to confluence and transfected siRNA by using Hiperfect Reagent (Takara) in Opti-MEM medium (Invitrogen). The sequence of SLCO4A1-AS1 targeting siRNA was: 5'-GCCTGAGCTTGTTCACAAA-3'. Sequences of non-target scramble controls were provided by Sangon Biotech Company (Shanghai, China).

### Cell proliferation assays

CCK-8 and clone formation assays were used to measure cell proliferative capacity. For the CCK-8 assay, cells were seeded into 96-well plates. At the indicated time point, 10 µl of CCK-8 solution was added to each well and incubated for 4 hours at 37 °C. Then, the absorbance at 450 nm was measured. For the clone formation assay, 2000 cells per well were seeded in 6-well plates and cultured for 2 weeks. Then colonies were fixed with 4% paraformaldehyde and stained with 2.5% crystal violet.

### Cell migration and invasion assay

Wound healing assays were used to examine CRC cell migration ability. Cells were seeded in six-well plates. After 24 hours of transfected siRNA, a vertical wound in the cell monolayer was generated through a 10 µl tip and washed three times with PBS to remove cell debris. The wound width was measured by a microscope at the designated time periods and the wound area was calculated using Image J.

Transwell analysis with Matrigel was used to measure tumor cell invasion capacity. Next, a total of 2×10^5^ cells in 100 µl of 2% FBS medium was added to the top of a transwell cell culture chamber (8 µm pore size, BD Biosciences, New Jersey, USA) coated with 50 µl Matrigel (BD Biosciences, USA), and 600 µl of 20% FBS containing medium was added to the lower chamber. The cells were incubated at 37 °C or 24 hours, and then, migrated tumor cells were fixed with 4% paraformaldehyde and stained with 2.5% crystal violet. Cells on the upper surface were wiped by a cotton bud. The number of invasive cells was counted from 6 randomly selected 100× fields under the microscope and shown as the average for average per field.

### Western blotting

For Western blot analysis, cells were lysed with RIP buffer (Boster) supplemented with RNase inhibitor and phosphatase inhibitor after cell transfection. Protein concentration was identified using a BCA kit (Invitrogen). The lysate sample was separated on a 10% SDS-PAGE gel and blotted onto blotted onto PVDF membranes. The membrane was incubated with primary antibody overnight at 4 °C, including EGFR (1:2000, Abcam), P-EGFR (1:2000, Abcam), KRAS (1:2000, Abcam), BRAF (1:2000, Abcam), MEK1/2 (1:2000, Abcam), P-MEK1/2 (1:1000, Proteintech), ERK (1:2000, Abcam), P-ERK (1:2000, Abcam), MAP3K1 (1:700, Proteintech), P-MAP3K1 (1:1000, Proteintech), β-actin (1:700, Proteintech), incubate for 2 hours at room temperature with anti-rabbit secondary antibody. Finally, protein band detection was performed using a Chemiluminescent Reagent (ECL) kit (Beyotime Biotechnology).

### Statistical analysis

All experiments were independently repeated at least triplicate. All statistical analyses were performed using Excel software version 2007 (Microsoft, USA) and performed using Graphpad prism 5 software. All data are represented as mean ± SEM and differences between the two independent groups were evaluated by Student's t-test. Overall survival (OS) was calculated using the Kaplan-Meier method, and the results of the analysis were considered significantly in a log-rank test if *P*<0.05. A two-tailed *P* value of 0.05 or less was considered statistically significant.

## Results

### SLCO4A1-AS1 is highly expressed in CRC

**Figure 1:**
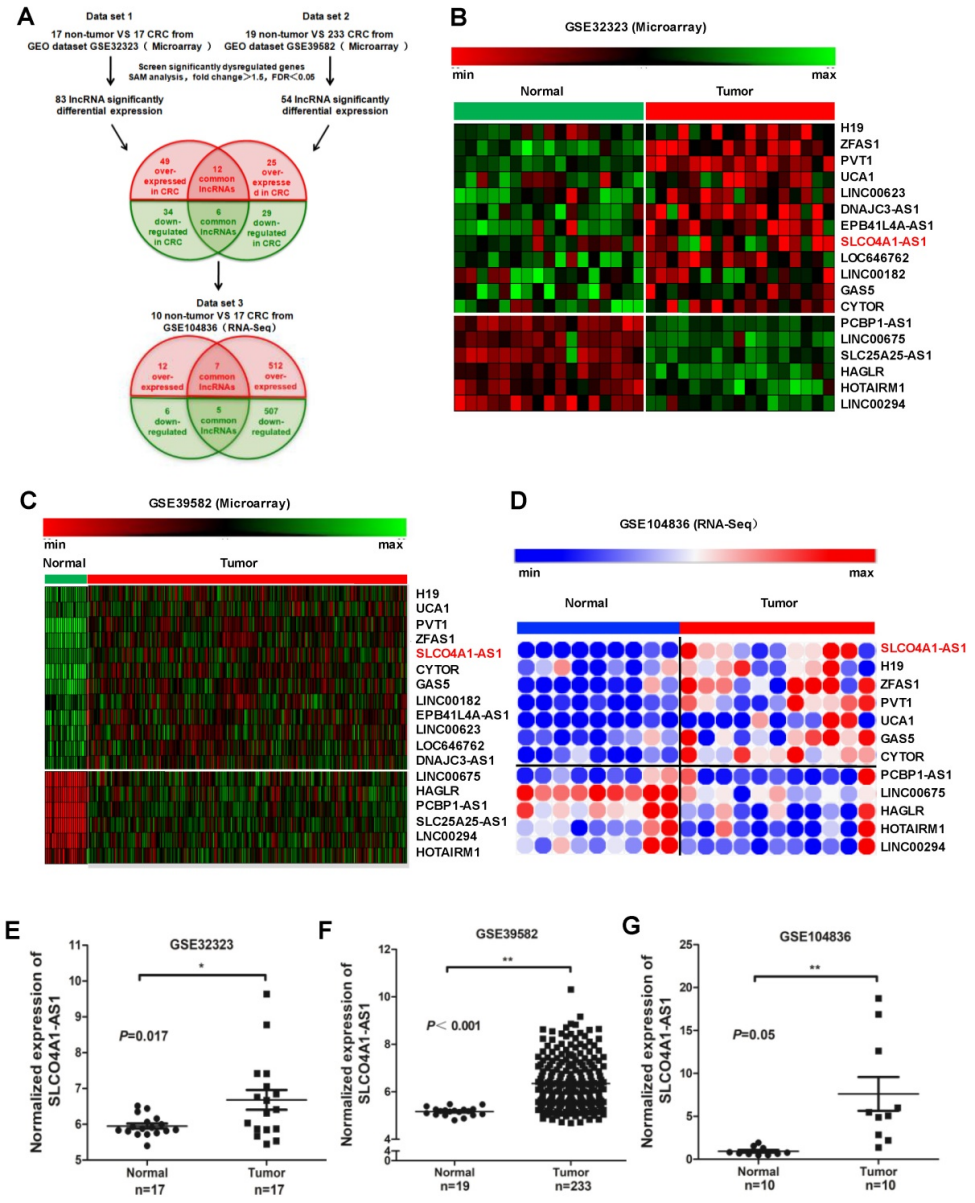
** SLCO4A1-AS1 is highly expressed in CRC. (A)** Schematic overview of the workflow used to identify dysregulated lncRNAs in two CRC microarray datasets (GSE32323, GSE39582), and one RNA-seq dataset (GSE104836). **(B-D)** Heatmap of overlapping dysregulated lncRNAs mined from the GEO data set. **(E-G)** SLCO4A1-AS1 expression, as measured by Affymetrix microarray, was upregulated in CRC tissues when compared with normal colorectal tissues in GSE32323, GSE39582, and GSE104836.

We downloaded two gene microarrays datasets GSE32323, GES39582, and RNA sequencing dataset GSE104836 from the GEO database to explore the differential expression of lncRNAs between CRC and normal tissues (Figure 1A). Through aggregation of differentially expressed lncRNAs from these two datasets of gene microarrays, 18 overlapping probsets, revealing 12 upregulated and 6 downregulated lncRNAs (Figure 1B and 1C). Further comparison with the GSE104836 dataset revealed 12 genes that were differentially expressed, including 7 upregulated and 5 downregulated lncRNAs (Figure 1A, 1D). SLCO4A1-AS1 expression was one of the most significantly upregulated in the CRC tissues compared to non-tumor tissues according to the GSE32323, GES39582, and GSE104836 datasets (Figure 1E-1G).

H19, ZFAS1, and PVT1, some famous oncogenic lncRNAs in many tumors, also listed and overexpressed among these overlapping probe sets^41^-^45^. Most were unknown or not well investigated in functions and mechanisms, such as SLCO4A1-AS1, LOC646762, LINC00182, and LINC00294. To investigate the role of lncRNAs in CRC, we focused on SLCO4A1-AS1 as a follow-up study, for which the expression was most significantly, and remained poorly investigated.

### Overexpression of SLCO4A1-AS1 predicts poor prognosis

**Figure 2:**
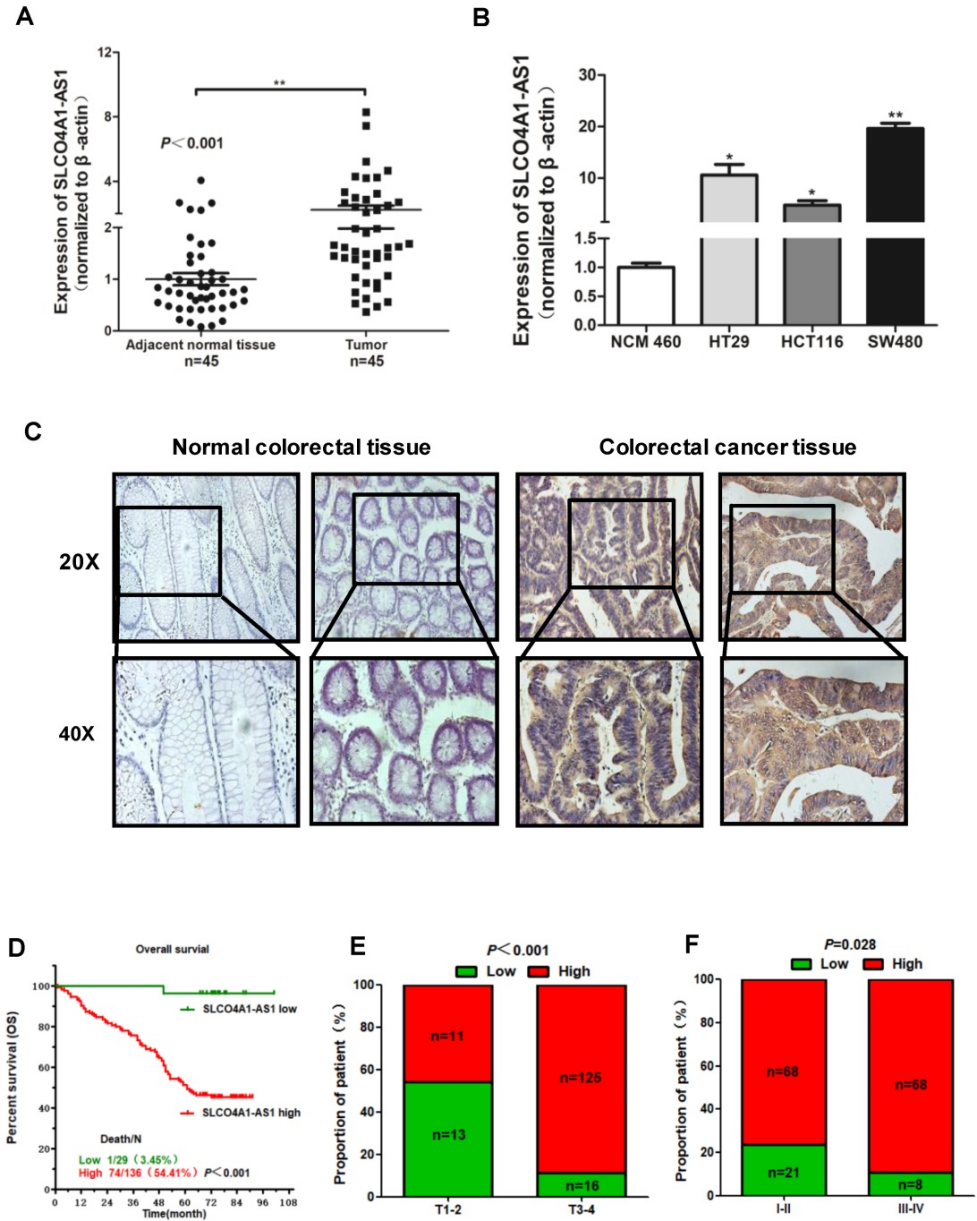
** Overexpression of SLCO4A1-AS1 predicts poor prognosis. (A)** SLCO4A1-AS1 expression was higher in CRC tissue samples (Tumor, n=45) than that in adjacent normal tissues (Adjacent normal tissues, n=45). **(B)** SLCO4A1-AS1 expression was significantly increased in CRC cell lines (HT29, HCT116, SW480) compared with NCM460, a normal colon cell line. **(C)** SLCO4A1-AS1 expression measured by *in situ* hybridization in paraffin embedded CRC biopsies. Upper panel: magnification=20×; lower panel: magnification=40×. **(D)** The highly expressed SLCO4A1-AS1 was correlated with shorter overall survival. **(E and F)** SLCO4A1-AS1 expression was associated with clinical stages.

To further verify the expression of SLCO4A1-AS1 in CRC, we collected 45 CRC tissues and 45 adjacent normal colorectal tissues detected the expression level of SLCO4A1-AS1 by RT-PCR. The results showed that SLCO4A1-AS1 was significantly upregulated in CRC tissues compared to adjacent normal colorectal tissues (Figure 2A, *P*=0.001). This is consistent with the results of the GEO dataset. In addition, SLCO4A1-AS1 was also overexpressed in three CRC cell lines HT29, HCT116, SW480 compared with NCM460, a normal colon cell line (Figure 2B, *P*<0.050, *P*<0.050, *P*<0.010).

We further used *in situ* hybridization to verify the expression of SLCO4A1-AS1 in colorectal cancer tissues and its correlation with clinical pathological parameters of CRC patients. The SLCO4A1-AS1 was highly expressed in the cancer nest of colorectal cancer tissue compared with normal colorectal tissue samples (Figure 2C). High expression of SLCO4A1-AS1 was also associated with shorter overall survivor of CRC patients, which indicates that upregulated SLCO4A1-AS1 could predict poor prognosis (Figure 2D, *P*<0.001). We then analyzed the association of SLCO4A1-AS1 expression with clinicopathological parameters, such as the level of local invasion (T stage), lymphatic invasion (N stage), and distant metastasis (M stage). The data indicated that the expression of SLCO4A1-AS1 was positively associated with the local invasion (T stage) (Figure 2E, *P*<0.001), and the TNM stage (Figure 2F, *P*=0.028). Detailed results of clinical parameters and expression were shown in Table 1. Moreover, the available follow-up data of therapy effects and SLCO4A1-AS1 expression were show in Table 2. The above results demonstrate that SLCO4A1-AS1 is highly expressed in CRC and is associated with higher local invasion, TNM stage, and poor prognosis.

### Knockdown of SLCO4A1-AS1 suppresses CRC cell proliferation

**Figure 3:**
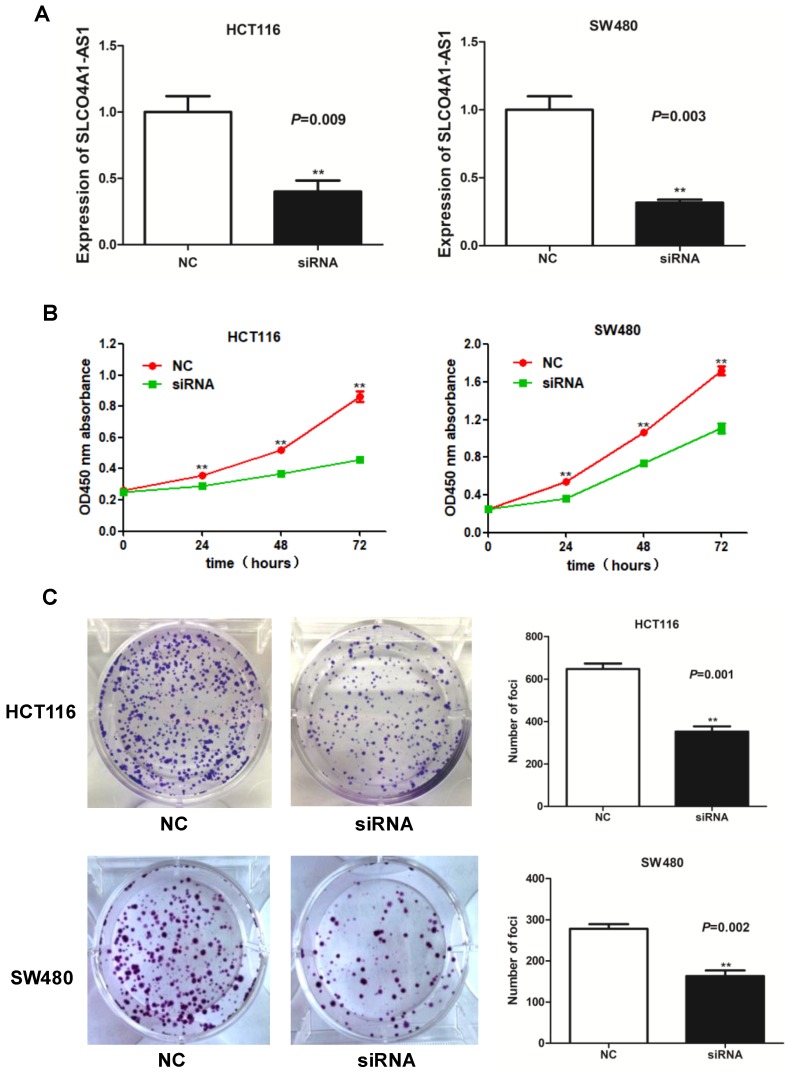
** Knockdown of SLCO4A1-AS1 suppresses CRC cell proliferation in vitro. (A)** siRNA dramatically suppressed SLCO4A1-AS1 expression in HCT116 and SW480 cells (*P*=0.009, *P*=0.003). **(B)** CCK-8 assay showed that knockdown of SLCO4A1-AS1 resulted in growth retardation of HCT116 and SW480 cells (*P*<0.001, *P*<0.001). **(C)** Clone formation assay shows that SLCO4A1-AS1 knockdown suppressed the proliferation of HCT116 and SW480 cells (*P*=0.001, *P*=0.002).

To investigate the function of lncRNA SLCO4A1-AS1 in CRC, we used siRNA to knockdown of SLCO4A1-AS1 expression in CRC cells. We knockdown the expression of SLCO4A1-AS1 in CRC by using short interfering RNA (siRNA) in CRC cell lines HCT116 and SW480. RT-PCR results showed that SLCO4A1-AS1 expression was significantly inhibited in HCT116 and SW480 cells after transfection of siRNA (Figure 3A, *P*=0.009, *P*=0.003). CCK-8 assay showed that knockdown of SLCO4A1-AS1 resulted in growth retardation of HCT116 and SW480 cells (Figure 3B, *P*<0.001, *P*<0.001). Similarly, the results of the colony formation assay were coincided with the CCK-8 results, and CRC cells knockdown of SLCO4A1-AS1 formed fewer colony colonies than the control group (Figure 3C, *P*=0.001, *P*=0.002).

### Knockdown of SLCO4A1-AS1 inhibits CRC cell migration and invasion

**Figure 4:**
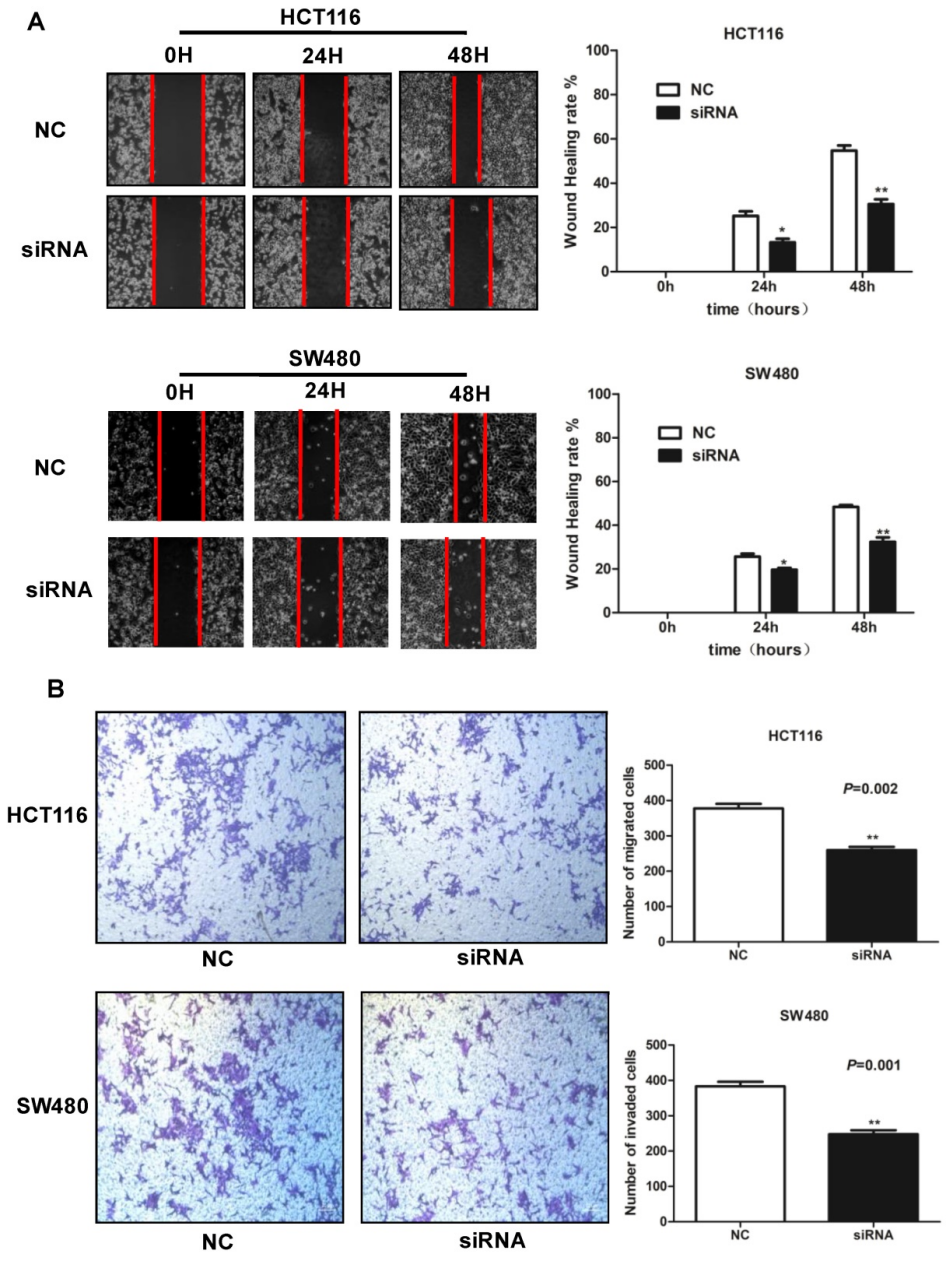
** Knockdown of SLCO4A1-AS1 inhibits CRC cell migration and invasion in vitro. (A and B)** HCT116 and SW480 cells were transfected with SLCO4A1-AS1 siRNA, or scramble siRNA. 24 hours after transfection, cells were subjected to a wound healing assays or transwell assays to measure migration (*P*<0.010, *P*<0.010) or invasive capacity (*P*=0.002, *P*=0.001).

To explore the roles of SLCO4A1-AS1 in CRC cell migration and invasion, we further performed wound healing assays and transwell assays. Wound healing assays showed that knockdown of SLCO4A1-AS1 significantly reduced the migration of HCT116 and SW480 cells compared to the control group (Figure 4A, *P*<0.010, *P*<0.010). Transwell assays showed that knockdown of SLCO4A1-AS1 significantly inhibited invasion of HCT116 and SW480 cells compared to the control group (Figure 4B, *P*=0.002, *P*=0.001). Therefore, the above results demonstrate that knockdown of SLCO4A1-AS1 can inhibit the migration and invasion of CRC cells, suggesting that the highly expressed SLCO4A1-AS1 functions as an oncogene in colorectal cancer.

### SLCO4A1-AS1 is associated with CRC via EGFR/MAPK signaling pathway

**Figure 5:**
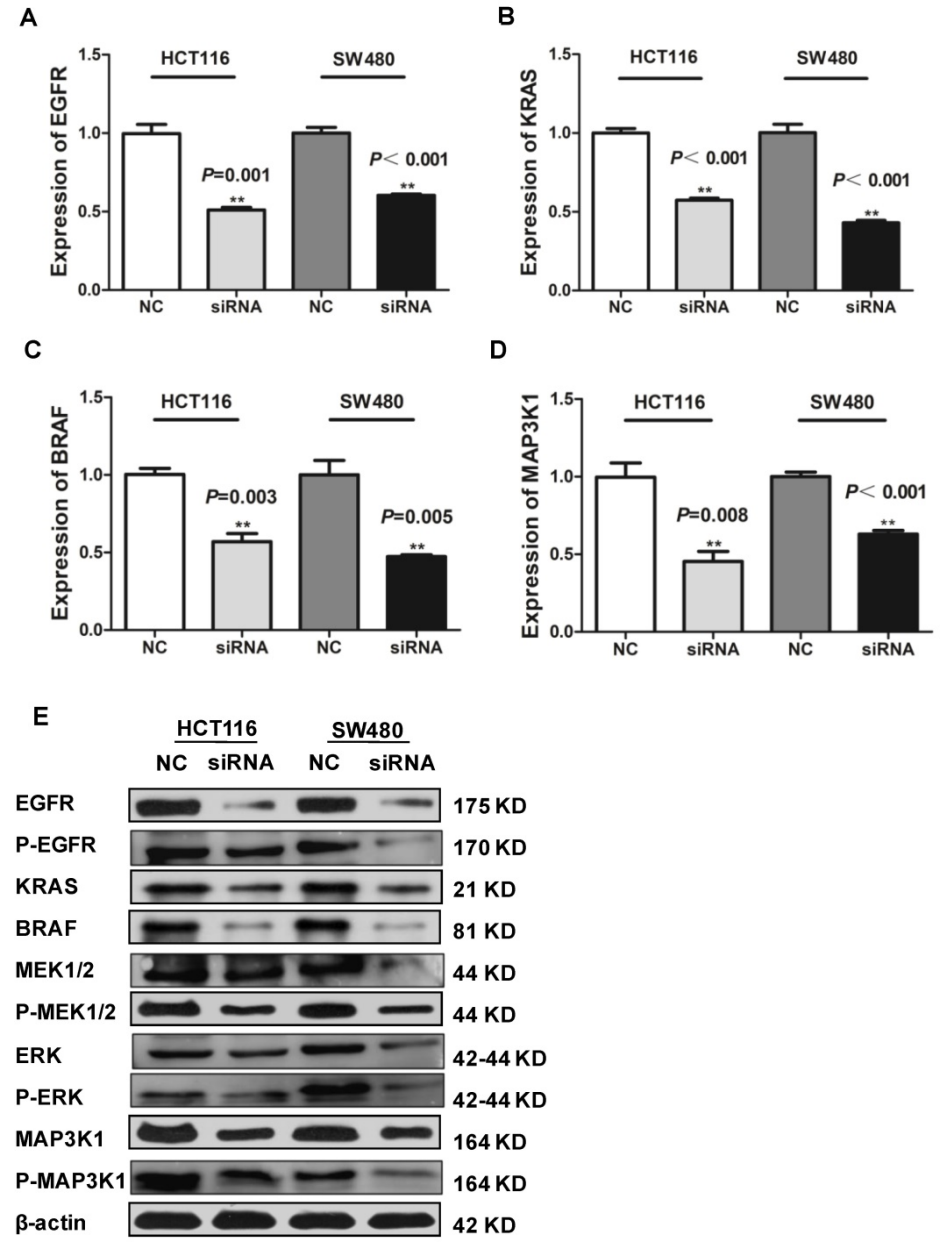
** Identification of SLCO4A1-AS1 regulated genes in EGFR/MAPK signaling pathway. (A-D)** mRNA levels of EGFR/MAPK pathway-associated proteins were detected by Q-PCR in HCT116 and SW480 cells transfected with negative control (NC) or SLCO4A1-AS1 siRNA.** (E)** Expression of EGFR/MAPK pathway-associated proteins and its corresponding phosphorylation status proteins levels were detected by western blot in HCT116 and SW480 cells transfected with NC or SLCO4A1-AS1 siRNA. β-actin was used as an internal control.

LncRNAs can affect the occurrence and development of CRC through a variety of signaling pathways. Many studies have shown that the EGFR/MAPK pathway is aberrantly activated in CRC^46^, which is thought to be responsible for cancer cell proliferation and metastasis. We knocked down SLCO4A1-AS1 in HCT116 and SW480 cells, and detected mRNA expression levels of EGFR、KRAS、BRAF an MAP3K1 by RT-PCR. The results showed that EGFR, KRAS, BRAF and MAP3K1 expression were downregulated after SLCO4A1-AS1 knockdown (Figure 5A, *P*=0.001, *P*<0.001; Figure 5B, *P*<0.001, *P*<0.001; Figure 5C, *P*=0.003, *P*=0.005; Figure 5D, *P*=0.008, *P*<0.001). In order to clarify the mechanisms of SLCO4A1-AS1 in CRC, the expression of EGFR, KRAS, BRAF, MEK, ERK, MAP3K1 protein and its corresponding phosphorylation status were analyzed by western blotting. The results were consistent with RT-PCR. The knockdown of SLCO4A1-AS1 could downregulate the expression of EGFR, KRAS, BRAF, MEK, ERK, MAP3K1 protein and its corresponding phosphorylation status (Figure 5E). Therefore, the above results indicated that SLCO4A1-AS1 may promote the progression of CRC through the EGFR/MAPK pathway.

## Discussion

Recent studies have shown that lncRNAs play an important role in tumor differentiation, proliferation, metastasis and other tumor development processes^15^,^47^,^48^. The pathogenesis and carcinogenic mechanisms of CRC are multifactorial and complex processes involving different genetic and epigenetic changes. LncRNA has been found to play an important role in the occurrence and development of CRC, including: APC^49^,^50^, OCC-1^33^, FEZF1-AS1^47^,^51^, SNHG5^52^,^53^ and so on.

In this study, we combined two GEO gene microarrays datasets to construct a differential expression profile of lncRNA, and combined the sequencing dataset to construct a common differential expression profile, and screened lncRNA SLCO4A1-AS1 for subsequent expression verification, functional exploration and molecular mechanism. High expression of SLCO4A1-AS1 in CRC is associated with the poor prognosis of CRC patients and as a potential biomarker. After siRNA-mediated silencing of SLCO4A1-AS1, we found that it significantly inhibited the proliferation, migration and invasion of CRC cells. Studies have shown that the EGFR/MAPK pathway is involved in cell proliferation, promote tumor development and play a key role in the development of CRC^54^-^56^. EGFR is abnormally expressed in cancer^57^. The MAPK pathway is one of the major downstream effectors of EGFR. The EGFR/MAPK pathway is aberrantly activated in CRC, which is thought to be responsible for cancer cell proliferation, migration, and invasion^55^.

Hyperactivation of the EGFR/MAPK signaling often leads to various cancers such as esophageal squamous cell carcinoma and CRC^58^,^59^. For instance, LINC01225 promotes occurrence and metastasis of hepatocellular carcinoma by binding to EGFR and increasing the protein level of EGFR, then activating EGFR/MAPK signaling pathway^60^. Additionally, activation of EGFR/MAPK signaling promotes CRC metastasis^61^.

In our study, we found that SLCO4A1-AS1 knockdown severely decreased the mRNA level and protein level of EGFR, KRAS, BRAF, MEK, ERK, MAP3K1 by the mechanism that inhibited the phosphorylation. LncRNAs may associate with proteins to regulate their stability, activity or other properties^62^. EGFR level plays a pivot role in the MAPK pathway^63^. The increasing of EGFR protein level may lead to CRC metastasis^64^. Regulation of EGFR/MAPK signaling is complicated and delicate. How lncRNAs interact with proteins on EGFR/MAPK pathways to fine-tune proliferation, migration and invasion of CRC requires further elucidation.

**Figure 6:**
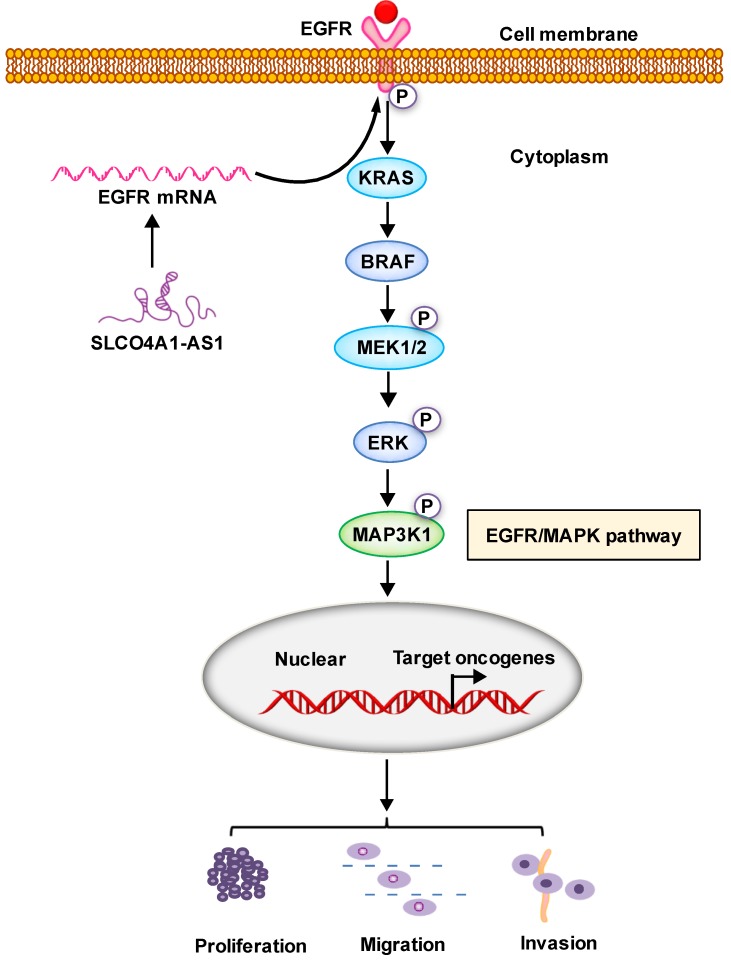
** Proposed schematic model illustrating the role of SLCO4A1-AS1 in regulating CRC by EGFR/MAPK signaling pathway.** SLCO4A1-AS1 influences EGFR/MAPK signaling pathway by promoting the expression of EGFR, KRAS, BRAF, MEK, ERK, MAP3K1 and its corresponding phosphorylated protein levels, which further affect the proliferation, migration and invasion of CRC cells.

In conclusion, our results suggest that SLCO4A1-AS1 may be involved in the development of CRC, which may play an important role in CRC tumorigenesis and may be a useful biomarker for predicting CRC prognosis. We contend that SLCO4A1-AS1 activates EGFR/MAPK pathway, facilitates the proliferation, migration and invasion of CRC cancer cells. SLCO4A1-AS1 may serve as potential targets for future treatment (Figure 6).

## Supplementary Material

Table S1.

## Figures and Tables

**Table 1:**
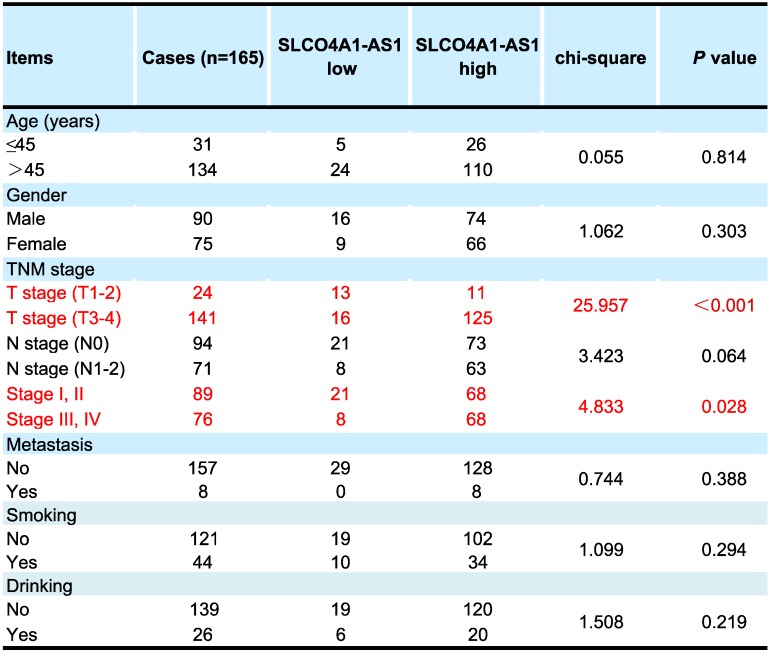
Correlation of clinical parameters with SLCO4A1-AS1 in CRC

**Table 2:**
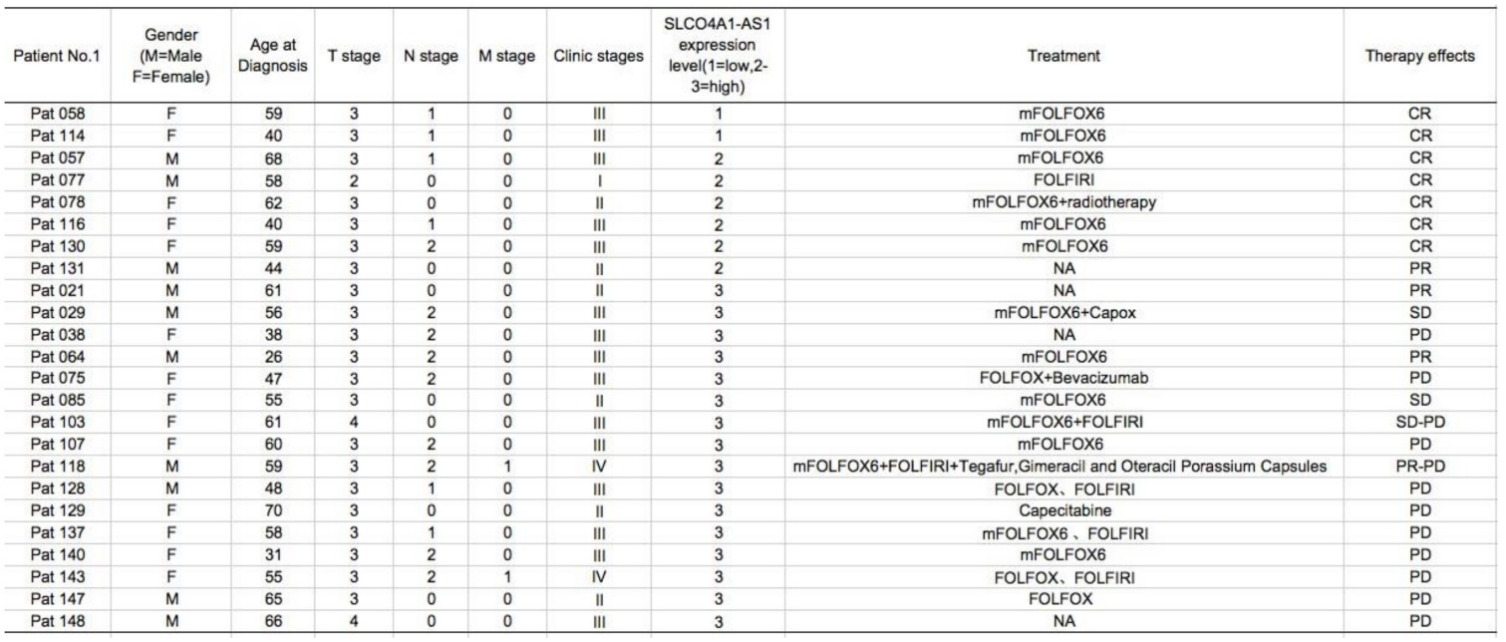
Therapy effects and expression levels of SLCO4A1-AS1
